# Vitamin D-Regulated MicroRNAs: Are They Protective Factors against Dengue Virus Infection?

**DOI:** 10.1155/2016/1016840

**Published:** 2016-05-11

**Authors:** John F. Arboleda, Silvio Urcuqui-Inchima

**Affiliations:** Grupo Inmunovirología, Facultad de Medicina, Universidad de Antioquia (UdeA), Calle 70 No. 52-51, Medellín, Colombia

## Abstract

Over the last few years, an increasing body of evidence has highlighted the critical participation of vitamin D in the regulation of proinflammatory responses and protection against many infectious pathogens, including viruses. The activity of vitamin D is associated with microRNAs, which are fine tuners of immune activation pathways and provide novel mechanisms to avoid the damage that arises from excessive inflammatory responses. Severe symptoms of an ongoing dengue virus infection and disease are strongly related to highly altered production of proinflammatory mediators, suggesting impairment in homeostatic mechanisms that control the host's immune response. Here, we discuss the possible implications of emerging studies anticipating the biological effects of vitamin D and microRNAs during the inflammatory response, and we attempt to extrapolate these findings to dengue virus infection and to their potential use for disease management strategies.

## 1. Introduction

Activation of innate immune cells results in the release of proinflammatory mediators to initiate a protective local response against invading pathogens [1]. However, overactivated inflammatory activity could be detrimental since it can cause tissue damage and even death of the host. Therefore, negative feedback mechanisms are required to control the duration and intensity of the inflammatory response [1, 2]. Although little is known about the molecular mechanisms occurring during dengue virus (DENV) infection/disease, it has been suggested that the immune response initiated against the virus greatly contributes to pathogenesis. Indeed, several symptoms of the disease are tightly related to imbalanced immune responses, particularly to high production of proinflammatory cytokines [3, 4] suggesting an impairment of homeostatic mechanisms that control inflammation. Interestingly, vitamin D has been described as an important modulator of immune responses to several pathogens and as a key factor enhancing immunoregulatory mechanisms that avoid the damage that arises from excessive inflammatory responses [5, 6], as in dengue disease [7]. Mounting evidence obtained from human populations and experimental in vitro studies has suggested that this hormone can play a key role in the immune system's response to several viruses [8–14], thereby becoming a potential target of intervention to combat DENV infection and disease progression. Among several mechanisms, vitamin D activity has been associated with the expression of certain microRNAs (miRs) [15] that are one of the main regulatory switches operating at the translational level [16]. miRs constitute approximately 1% of the human genome and their sequences can be found within introns of other genes or can be encoded independently and transcribed in a similar fashion to mRNAs encoded by protein-coding genes [16]. A typical mature miR of 18–23 base pairs associates with the RNA-induced silencing complex (RISC) and moves towards the target mRNA [17]. Once there, the miR binds to the complementary sequence in the 3′untranslated region (3′UTR) of the mRNA, thereby inducing gene silencing through mRNA cleavage, translational repression, or deadenylation [16]. A single miR may directly regulate the expression of hundreds of mRNAs at once and several miRs can also target the same mRNA resulting in enhanced translation inhibition [18]. Targeting of specific genes involved in modulation of immune response pathways by miRs provides a finely tuned regulatory mechanism for the restoration of the host's resting inflammation state [19–21]. Since the association between vitamin D and miR activity may play a relevant role in ongoing DENV infections, here we provide an overview of DENV-induced inflammatory responses and the early evidence anticipating a possible participation of the vitamin D and miR interplay regulating antiviral and inflammatory responses during DENV infection/disease.

## 2. DENV and the Immune Response

DENV is an icosahedral-enveloped virus with a positive sense single-stranded RNA (ssRNA) genome that belongs to the family Flaviviridae, genus* Flavivirus*. There are four phylogenetically related but antigenically distinct viral serotypes (DENV 1–4) able to cause the full spectrum of the disease [22]. In addition, a sylvatic serotype (DENV-5), with no evidence regarding its ability to infect humans, has been recently reported [23]. DENV is transmitted by* Aedes* mosquitoes in tropical and subtropical areas where the disease has become a major public health threat and one of the most rapidly spreading vector-borne diseases in the world, with an increasing incidence of 30-fold in the past 50 years [24, 25]. An estimated 3.6 billion people live in high risk areas worldwide and it is estimated that over 390 million cases occur every year, of which 96 million suffer from dengue fever [26–28]. Although only a minor number of cases may progress to the severe forms of the disease, 21.000 deaths are reported annually [27]. Guidelines of the World Health Organization (WHO) recognize dengue as a clinical continuum from dengue fever (DF), a nonspecific febrile illness, to dengue with or without warning signs that can progress to dengue hemorrhagic fever (DHF) or dengue shock syndrome (DSS) [3]. These severe forms of the disease are characterized by a wide spectrum of symptoms, including the development of vascular permeability, plasma leakage, thrombocytopenia, focal or generalized hemorrhages, and tissue and/or organ damage that may lead to shock and death [29, 30]. Besides ecoepidemiology, host genetic variations, and virus virulence, the risk factor is increased mainly by secondary infections with different dengue serotypes, presumably through a mechanism known as antibody-dependent immune enhancement (ADE), whereby nonneutralizing antibodies from previous heterotypic infections enhance virus entry via receptors for immunoglobulins or Fc receptors (FcRs) [29, 31, 32].

**Figure 1:**
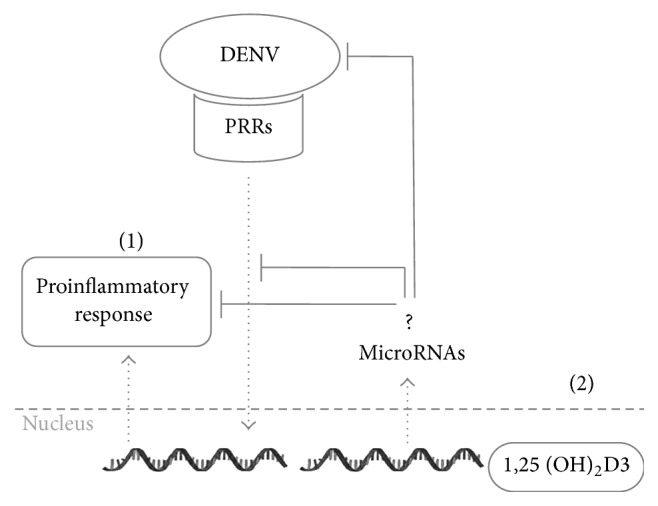
Potential link between vitamin D and miR controlling DENV-induced inflammatory response and antiviral activity. (1) DENV replication products and proteins are recognized by several PRRs whose signaling pathways promote the proinflammatory response. (2) Vitamin D activity induces transcription of microRNAs and other target genes that play a critical role in the control of inflammation-related signaling pathways and antiviral activity.

Skin is the first barrier for the invading DENV and the site where innate immunity exerts the first line of defense [33]. Following the bite by an infected mosquito, local tissue resident dendritic cells (DCs) and macrophages are the main targets of the virus [34, 35]. The viral structural E protein binds to cellular receptors, such as DC-SIGN (Dendritic Cell-Specific Intercellular adhesion molecule-3-Grabbing Nonintegrin), CLEC5A (C-type lectin domain family 5, member A), and MR (mannose receptor), allowing internalization of the virus through receptor-mediated endocytosis [22, 36–38]. Once in the cytoplasm, DENV replication products, such as double-stranded RNA (dsRNA) or genomic ssRNA, are sensed by several pattern recognition receptors (PRRs) (Figure 1), including TLR3, TLR7, TLR8, the cytosolic receptors RIG-I (Retinoic acid Inducible Gene-1), and MDA-5 (Melanoma Differentiation-Associated protein 5) [39–43]. Subsequently, this subset of PRRs triggers the activation of intracellular pathways, leading to the activation of transcription factors such as interferon regulatory factors 3 and 7 (IRF3 and IRF7) and the Nuclear Factor *κ*B (NF-*κ*B) and the later production of type I interferons and proinflammatory cytokines promoting an antiviral response [44, 45]. Additionally, the local activation of natural killer (NK) cells, neutrophils, and mast cells by the presence of the virus induces more proinflammatory mediators, complement activation, and the commitment of cellular and humoral immune responses to clear and control viral infection [46].

### 2.1. Inflammation and Cytokine Storm

Although the immune response is critical to combat and overcome invading pathogens, it is believed that the immune response greatly contributes to progression of dengue disease [31]. The pathogenesis and progression to the severe forms of dengue are still not completely understood; however, most cases are characterized by bleeding, hemorrhage, and plasma leakage that can progress to shock or organ failure [87, 88]. These physiological events are preceded by a hyperpermeability syndrome caused mainly by an imbalance between proinflammatory and anti-inflammatory cytokines produced in response to virus infection. The predominant proinflammatory mediators or “cytokine storm,” secreted mainly by T cells, monocytes/macrophages, and endothelial cells (Table 1), promotes endothelial dysfunction by generating an endothelial “sieve” effect that leads to fluid and protein leakage. Increasing evidence suggests that endothelial integrity and vascular permeability are affected by proinflammatory cytokines through the induction of apoptosis and the modulation of tight junction molecules within endothelial cells [47, 52, 89, 90]. In addition, it has also been reported that these cytokines may often have synergistic effects and may induce expression of other cytokines, generating a positive feedback mechanism leading to further imbalanced levels of inflammatory mediators and higher permeability [4].

This oversustained inflammatory response may be due to an impairment of the regulatory mechanisms that control the duration and intensity of inflammation or cytokine production, especially through the regulation of PRR signaling activation [20]. Several studies have shown that alterations in proinflammatory cytokine production during DENV infection/disease can be attributed to variations in recognition and activation of TLR signaling, which contributes to progression of the disease (Figure 1) [91, 92]. It was recently reported that DENV NS1 proteins may be recognized by TLR2, TLR4, and TLR6 enhancing the production of proinflammatory cytokines and triggering the endothelial permeability that leads to vascular leakage [93, 94]. Interestingly, our group has recently shown a differential expression of TLRs in dendritic cells (DCs) of dengue patients depending on the severity of the disease [95]. Indeed, there was an increased expression of TLR3 and TLR9 in DCs of patients with DF in contrast to a poor stimulation of both receptors in DCs of patients with DHF. Conversely, a lower expression of TLR2 in DF patients compared to DHF patients was also observed. Additionally, IFN-*α* production was also altered via TLR9, suggesting that DENV may affect the type I IFN response through this signaling pathway [95]. Indeed, DENV has successfully evolved to overcome host immune responses, by efficiently subverting the IFN pathway and inhibiting different steps of the immune response through the expression of viral nonstructural proteins that antagonize several molecules of this activation pathway [96, 97]. Although DENV may evade immune recognition [42], cumulative data have shown that it is sensed by both TLR3 and TLR7/8 and activates signaling pathways upregulating IFN-*α*/*β*, TNF-*α*, human defensin 5 (HD5), and human *β* defensin 2 (H*β*D2) [39–41]. In addition, RIG-I and MDA-5 are also activated upon DENV infection and are essential for host defense against the virus [40]. Moreover, TLR3 controls DENV2 replication through NF-*κ*B activation, suggesting that TLR3 agonists such as Poly (I : C) (Polyinosinic : Polycytidylic Acid) might work as immunomodulators of DENV infection [39]. Furthermore, besides DENV recognition and binding, C-type lectins such as the mannose receptor (MR) and CLEC5A may contribute to the inflammatory responses [98–100]. CLEC5A plays a critical role in the induction of NLRP3 inflammasome activation during DENV infection and enhances the release of IL-18 and IL-1*β* that are critical for activation of Th17 helper cells [99, 101].

While innate immune activation and proinflammatory cytokine production are being investigated during the course of DENV infections [53, 92, 102], vitamin D activity has gained special attention due to its importance in the modulation of the innate response. An increasing number of reports suggest that vitamin D activity is associated with the modulation of components implicated in antiviral immune responses and in the regulation of proinflammatory cytokine production through the modulation of miR expression [6, 13, 15, 103]. Although there is little information from observational studies and clinical trials demonstrating the role of vitamin D during dengue virus infection, here we postulate a potential role of vitamin D controlling progression of dengue disease and provide evidence of some vitamin D molecular mechanisms in support of our hypothesis.

## 3. Vitamin D: Antiviral and Anti-Inflammatory Activity

In addition to its well-known role in bone mineralization and calcium homeostasis, vitamin D is recognized as a pluripotent regulator of biological and immune functions [104]. A growing body of evidence suggests that it plays a major role during the immune system's response to microbial infection, thereby becoming a potential intervener to control viral infections and inflammation [13, 105, 106]. The term vitamin D refers collectively to the active form 1*α*-25-dihydroxyvitamin D_3_ [1*α*-25(OH)_2_D3] and the inactive form 25-hydroxyvitamin D_3_ [25(OH)D_3_] [107]. For their transport within the serum, vitamin D compounds bind to the vitamin D binding protein (DBP) and this complex is recognized by megalin and cubilin (members of low-density lipoprotein receptor family) that then internalize the complex by invagination [108]. Intracellular trafficking of vitamin D metabolites to specific destinations is performed by members of the HSP- (Heat Shock Proteins-) 70 family [104]. In addition, vitamin D metabolites are also lipophilic molecules that can easily penetrate cell membranes and translocate to the nucleus, where 1*α*-25(OH)_2_D_3_ binds to the vitamin D receptor (VDR), thereby inducing heterodimerization of VDR with an isoform of the retinoid X receptor (RXR) [109]. The VDR-RXR heterodimer binds to vitamin D response elements (VDRE) present in the promoter of hundreds of target genes, whose products play key roles in cellular metabolism, bone mineralization, cell growth, differentiation, and control of inflammation (Figure 1) [104, 110, 111]. Besides VDR, other related vitamin D metabolic components such as the hydrolase CYP27B1, the enzyme that catalyzes the synthesis of active 1*α*-25-dihydroxyvitamin D_3_ from 25-hydroxyvitamin D_3_, are present and induced in some cells of the immune system during immune responses [112]. Thus, an increasing number of studies have explored the relationship between vitamin D activity and the immune system, specifically, the mechanisms whereby vitamin D exerts its antimicrobial and immunoregulatory activity [14, 113, 114]. Here, we highlight those modulating antiviral and inflammatory responses.

Although controversial data have been reported, increasing clinical and observational studies have provided evidence supporting the protective features of vitamin D in viral infections, especially viral respiratory infections and HIV [13, 115, 116]. The activity of vitamin D in the innate immune system begins at the forefront of the body's defense against pathogens, the skin. Regardless of global serum vitamin D levels, sensing of microbial pathogens via PRRs induces upregulation of CYP27B1 and, as a consequence, local conversion of 1,25(OH)_2_D_3_ from 25(OH)D_3_, enhancing VDR nuclear translocation and subsequent transcription of target genes to exert antimicrobial effects [113, 117–119]. This establishes a linkage between vitamin D status and the intracrine and paracrine modulation of cellular immune responses, in which VDR and CYP27B1 activity are of central importance [117, 118, 120]. Indeed, this link is also evidenced by studies in which pathogen susceptibility associated with vitamin D deficiency/insufficiency levels is reduced by correct supplementation [121, 122]. Furthermore, some vitamin D-induced antiviral mechanisms have been shown by preliminary reports (Table 2). Peptides such as cathelicidins are strongly upregulated by 1,25(OH)_2_D_3_ due to its VDR response elements. In humans, active cathelicidin is known as LL-37 and has a C-terminal cationic antimicrobial domain that can induce bacterial membrane disruption and inhibition of herpes simplex virus, influenza virus, and retroviral replication, among others [55–57]. In fact, very recent reports have suggested an association between vitamin D and the LL-37 antiviral activity to HIV and rhinovirus [58, 59]. Likewise, HBD-2 is also induced by 1,25(OH)_2_D_3_. Interestingly, a correlation between VDR and HBD-2 was found to be associated with natural resistance to HIV infection, suggesting the potential participation of vitamin D-induced resistance to the virus [60, 106]. Moreover, vitamin D can also induce reactive oxygen species (ROS) that associates with suppression of the replicative activity of some viruses, such as hepatitis C virus (HCV) [61]. Although the vitamin D-induced antiviral mechanisms are not fully elucidated and further studies are needed to fully understand their roles, many are possible due to the pleiotropic nature of vitamin D and the complex transcriptional modulation of hundreds of genes controlled by its activity.

Several studies have reported a link between VDR polymorphisms and severe outcomes of bronchiolitis and acute lower respiratory tract infections (RTIs) with respiratory syncytial virus (RSV) [105]. Indeed, vitamin D supplementation is associated with reduced RTI, vitamin D status, and serum concentrations in children [123]. Likewise, some vitamin D supplementation studies have reported a reduction in cold/influenza linked to seasonal sunlight exposure and skin pigmentation [124]. In HIV infection, associations have also been reported between vitamin D levels with progression of the disease, survival times of HIV patients, CD4^+^ T cell counts, inflammatory responses, and potential impact of HAART (Highly Active Anti-Retroviral Therapy) treatments [125]. Finally, similar population and ecoepidemiological reports have associated the role of vitamin D in several viral infections, including DENV and other flaviviruses [10–13], not only highlighting inhibition of viral replication but also controlling the inflammatory response and progression of the disease.

In addition to viral control, vitamin D-induced immune mechanisms have important effects providing potential feedback modulation in pathways that regulate immune activation, avoiding excessive elaboration of the inflammatory responses and its potential risk for tissue homeostasis (Table 3) [5, 6, 126]. TLRs can both affect and be affected by VDR signaling and likewise some antimicrobial peptides associated with TLRs have demonstrated antiviral effects [6, 13, 127]. In this sense, and due to the interest in the modulatory effect of vitamin D on TLR expression and proinflammatory cytokine production, some authors have shown that vitamin D can induce hyporesponsiveness to PAMPs (Pathogen-Associated Molecular Patterns) by downregulating the expression of TLR2 and TLR4 on monocytes that in turn have been associated with impaired production of TNF-*α*, suggesting a critical role of vitamin D in regulating TLR driven inflammation [71]. Importantly, a link between the DENV NS1 protein and activation of the inflammatory response via TLR2 and TLR4 impacting the progression of the disease has very recently been described [93, 128]. DENV NS1 antigens may induce the activation of TLR2 and TLR4 inducing high secretion of proinflammatory mediators that enhance endothelial dysfunction and permeability [46, 94, 129, 130]. Interestingly, it was reported that 1,25(OH)_2_D_3_ significantly reduces the levels of TLR2/TLR4 expression and of proinflammatory cytokines (TNF-*α*, IL-6, IL-12p70, and IL-1*β*) produced by U937 cells after exposure to DENV [72]. The same approach used in primary human monocytes and macrophages led to similar results, consistent with data obtained in our laboratory [19]. It has been suggested that vitamin D may regulate proinflammatory cytokine levels by targeting TLR activation signaling molecules (Figure 1). Indeed, it has been reported that treatment of monocytes with 1,25(OH)_2_D_3_ regulates TLR expression via the NF-*κ*B pathway and reduces signaling of the mitogen-activated protein kinases MAPKs/p38 and p42/44 [19]. One of the most critical steps in NF-*κ*B regulation is I*κ*B*α* proteasomal degradation mediated by IKK (I kappa B Kinase) that leads to the nuclear entry of the NF-*κ*B heterodimer p65/p50 to transactivate gene expression, resulting in a decrease of inflammatory genes. Accordingly, a novel molecular mechanism has recently been described in which 1,25(OH)_2_D_3_ binding to VDR attenuates NF-*κ*B activation by directly interacting with the IKK*β* protein to block its activity and, consequently, the NF-*κ*B-dependent inflammatory response [76]. Besides TLR2 and TLR4, it has been shown that vitamin D can also downregulate the intracellular TLR9 expression and, subsequently, lead to less secretion of IL-6 in response to TLR9 stimulation [77]. Although intracellular downregulation of some PRRs such as TLR3, TLR7/8, and RIG-I/MDA5 may affect the potential antiviral response induced by type I IFN, various reports have shown that vitamin D treatment does not affect the type I IFN-induced antiviral response against various viruses [69, 131, 132]. In fact, it has been reported that porcine rotavirus (PRV) infection induces CYP27B1-dependent generation of 1,25(OH)_2_D_3_ which leads to an increased expression of TLR3 and RIG-I that consequently enhance the type I IFN-dependent antiviral response [76].

### 3.1. Vitamin D and miRs: Potential Implications for Inflammation Balance

Although vitamin D may impact distinct pathways and molecules to modulate inflammatory responses, current evidence suggests TLRs and TLR signaling mediators as main targets by which vitamin D modulates inflammation (Table 3) [6, 113, 133, 134]. However, a novel regulatory vitamin D mechanism in which TLR signaling/activation and miR function are associated has been recently documented, suggesting a crucial role of vitamin D and miRs for the host immune system homeostasis [15, 135, 136]. The participation of miRs as general regulatory mechanisms of initiation, propagation, and resolution of immune responses has been widely reviewed elsewhere [21, 137, 138]. Therefore, we discuss here its potential relationship with vitamin D activity in the control of inflammatory responses, attempting to extrapolate these findings to DENV infection.

The ability of vitamin D to regulate miRs and their emerging relationship have been proposed by means of several experimental and clinical approaches; however, the implications of their impact on inflammatory responses have only been studied in in vitro models [15, 20, 135, 136, 139]. In patient trials with vitamin D supplementation, significant differences in miR expression profiles have been reported, suggesting that dietary vitamin D may also globally regulate miR levels [15]. Although several mechanisms may be involved in regulating such a global effect, some authors have found that chromatin states may be altered by VDR activity, determining accessibility for binding of the transcription and regulation of activation or inhibition of transcription [140, 141]. This in turn could be of relevance for canonical VDR-VDRE-mediated transcription regulation. In fact, VDR-induced regulation of miRs via VDRE has been demonstrated for some miRs such as miR-182 and let-7a whose pri-miRs (Primary miR) have multiple VDR/RXR binding sites, suggesting that these miRs could potentially be regulated by vitamin D metabolites [67, 142]. Moreover, a negative feedback loop between some miRNAs and VDR signaling has been reported. This is the case of miR-125b whose overexpression can reduce VDR/RXR protein levels. Since miR-125b is commonly downregulated in cancer cells, it has been proposed that such a decrease in miR-125b may result in the upregulation of VDR and in increasing antitumor effects driven by vitamin D in cancer cell models [136].

Additionally, it has been reported that VDR signaling may attenuate TLR-mediated inflammation by enhancing a negative feedback inhibition mechanism (Figure 1). A recent report has shown that VDR inactivation leads to a hyperinflammatory response in LPS-cultured mice macrophages through overproduction of miR-155 which in turns downregulates the suppressor of the cytokine signaling (SOCS) family of proteins that are key components of the negative feedback loop regulating the intensity, duration, and quality of cytokine signaling [2, 143, 144]. As feedback inhibitors of inflammation, SOCS proteins are upregulated by inflammatory cytokines, and, in turn, they block cytokine signaling by targeting the JAK/STAT (Janus Kinase/Signal Transducer and Activator of Transcription) pathway [2]. Evidence suggests that SOCS inhibits the proinflammatory pathways of cytokines such as TNF-*α*, IL-6, and IFN-*γ* and can inhibit the LPS-induced inflammatory response by directly blocking TLR4 signaling by targeting the IL-1R-associated kinases (IRAK) 1 and 4 [20, 144]. Consequently, deletion of miR-155 attenuates 1,25(OH)_2_D_3_ suppression of LPS-induced inflammation, confirming that vitamin D stimulates SOCS1 by downregulating miR-155 [20]. Taken together, these results highlight the importance of the VDR pathways controlling the inflammatory response by modulating miRNA-155-SOCS1 interactions. Finally, an additional reinforcing issue that may validate the link between vitamin D activity and miRs is the fact that 1,25(OH)_2_D_3_ deficiency has been related to reduced leukotriene synthetic capacity in macrophages [145, 146]. Recently, it was reported that leukotriene B4 (LTB_4_) can upregulate macrophage MyD88 (Myeloid Differentiation primary response-88) expression by decreasing SOCS-1 stability that is associated with the expression of proinflammatory miRs, such as miR-155, miR-146b, and miR-125b, and TLR4 activation in macrophages [147]. miR-146 has been also shown as a modulator of inflammatory responses mediated by TLR4/NF-*κ*B and TNF-*α* [70]. Importantly, this miR has been found downregulated in patients with autoimmune disorders in which low levels of vitamin D have also been reported [148, 149]. These results suggest that vitamin D can orchestrate miR diversity involved in TLR signaling, thereby regulating inflammatory responses and activation of immune responses.

## 4. Insights into Vitamin D and DENV Infection

Little is known about the link between DENV infection and vitamin D; however, since severe dengue is associated with imbalanced production of proinflammatory cytokines, it is very tempting to suggest that vitamin D could play an important role in modulating the inflammatory responses during ongoing DENV infections. Although only few studies can illustrate a link between vitamin D activity and DENV infection or disease, these reports have provided preliminary epidemiological evidence supporting this novel hypothesis. Initially, it was reported that heterozygosity in the VDR gene was correlated with progression of dengue. It was shown in a small Vietnamese population where dengue is endemic that the low frequency of a dimorphic (T/t) “t” allele in the VDR gene was associated with dengue disease severity, suggesting a protective role of VDR activity against dengue disease progression [12]. Variations in VDR have also been associated with susceptibility to osteoporosis in humans and with reduced risk of tuberculosis and persistent hepatitis B virus infections [150–152], highlighting the importance of VDR variations in signaling and immune protection. Accordingly, a study revealed the association of the “T” allele with DHF, by showing that the “T” allele codes for a longer length VDR that is the least active form of VDR. Since vitamin D is known to suppress TNF-*α*, it is possible that such inappropriate VDR signaling may contribute to higher levels of inflammation, enhancing the susceptibility to severity of the disease [10]. Although the modulatory effect of vitamin D during DENV infection and disease has not been widely tested in human populations, initial studies have associated the effect of oral 25(OH)D_3_ supplementation with antiviral responses, resistance, and overcoming of the disease. Specifically, a study reported the case of five DF patients that ameliorated the signs and symptoms of the disease, improving the overall clinical conditions and reducing the risk of disease progression [11]. Interestingly, this may be linked to other clinical approaches where oral supplementation with vitamin D enhanced the antiviral response to HCV [63], another RNA virus belonging also to the family Flaviviridae.

The potential antiviral mechanism of vitamin D against DENV has yet not been fully explored; however, certain reports support the proposal that vitamin D could perform anti-DENV effects and immunoregulatory functions on innate immune responses [10–12]. In line with this, the effect of vitamin D treatment of human monocytic cell lines on DENV infection was recently reported [72]. The authors showed that cell exposure to 1,25(OH)_2_D_3_ resulted in a significant reduction of DENV-infected cells, a variable modulation of TLR2 and TLR4, and reduced levels of secreted proinflammatory cytokines such as TNF-*α*, IL-6, and IL-1*β* after infection [72]. The molecular mechanisms by which vitamin D can elicit an antiviral and anti-inflammatory role towards DENV have not been fully described, and although we observed that monocyte-derived macrophages differentiated in the presence of 1,25(OH)_2_D_3_ are less susceptible to DENV infection and express lower levels of mannose receptor restricting binding of DENV to target cells (manuscript in preparation), further studies are required to confirm that vitamin D treatment confers both anti-inflammatory and antiviral responses. Another interesting mechanism that could support the antiviral activity of vitamin D is the VDR-induced regulation of miRs via VDRE. This has been demonstrated for some miRs, such as let-7a (Table 2), whose pri-miR has multiple VDR/RXR binding sites that could potentially be regulated by vitamin D [67, 142]. miR let-7a belongs to a highly conserved family of miRs that contains other miRs previously reported to inhibit DENV replicative activity, such as let-7c [68]. Besides the members of the let-7 family, other miRs have also been associated with suppression of DENV infection and the inflammatory responses against the virus, as discussed below.

### 4.1. MicroRNAs in DENV Infection

Viruses strictly depend on cellular mechanisms for their replication; therefore, there is an obligatory interaction between the virus and the host RNA silencing machinery. Although virus-derived small interfering RNAs may induce changes in cellular mRNA and miR expression profiles to induce replication, cellular miRs can also target viral sequences or induce antiviral protein expression to inhibit viral replication and translation [153]. Indeed, during DENV infection, several cellular miRs have been reported to have an effect on the replicative activity of the virus and the permissiveness of the host cells. Although some host miRs can also enhance DENV replication [81, 154], here we highlight the miRs affecting DENV replicative activity and modulating the immune response (Table 4).

The expression levels of different miRs regulated during DENV infection have been screened in the hepatic cell line Huh-7. This approach identified miR let-7c as a key regulator of the viral replicative cycle that affects viral replication and the oxidative stress immune response through the protein Heme Oxygenase-1 (HO-1) by activating its transcription factor BACH1 (Basic Leucine Zipper Transcription Factor-1) [68]. In addition, it was recently reported that, after DENV-2 infection of the C6/36 cell line, endogenous miR-252 is highly induced and associated with a decreased level of viral RNA copies. This antiviral effect was explained by the fact that miR-252 targets the DENV-2 E protein gene sequence, downregulating its expression and therefore acting as an antiviral regulator [78]. Although DENV can escape the immune system by decreasing the production of type I IFN due to DENV NS5 and NS4B activity [42, 97], DENV infection also induces the upregulation of the cellular miR-30e^*∗*^ that suppresses DENV replication by increasing IFN-*β* production. This antiviral effect of miR-30e^*∗*^ depends mainly on NF-*κ*B activation by targeting the NF-*κ*B inhibitor I*κ*B*α* in DENV-permissive cells [79]. This antiviral effect induced by signaling of type I IFN is also promoted by miR-155 that has been reported to control virus-induced immune responses in models of infection with other members of the* Flavivirus* genus such as HCV [155–157]. In this latter model, the antiviral effect greatly depended on miR-155 targeting SOCS-1. This observation is in accordance with a study in which elevated expression of miR-150 in patients with DHF was correlated with suppression of SOCS-1 expression in monocytes [80] that in turn could be linked to the fact that vitamin D controls inflammatory responses through modulation of SOCS by downregulating miR-155 [20].

Although it has remained unclear whether endogenous miRs can interfere with viral replicative activity by targeting DENV sequences or viral mRNAs, some experimental approaches have shown the importance of miRs in restricting viral replication through this mechanism [85, 158–160]. Some artificial miRs (amiRs) have been described as targeting the highly conserved regions of the DENV-2 genome and promoting efficient inhibition of virus replication [158]. Using DENV subgenomic replicons carrying the specific miR recognition element (MRE) for miR-122 in the 3′-UTR of the DENV genome/mRNA, some authors have shown that the liver-specific miR-122 suppresses translation and replication of DENV by targeting this MRE sequence [81]. Likewise, the insertion of the MRE for the hematopoietic specific miR-142 into the DENV-2 genome restricts replication of the virus in DCs and macrophages, highlighting the importance of this hematopoietic miR in dissemination of the virus [82]. In addition, DENV replication is enhanced by the interaction of the viral genome 3′-UTR and the host polypyrimidine tract binding (PTB) protein that translocates from the nucleus to the cytoplasm facilitating DENV replication [36, 161, 162]. However, the PTB mRNA 3′-UTR contains MREs that can be targeted by miR-133a, providing a mechanism for the downregulation of the PTB protein expression levels [163]. Moreover, in our group, we found that miR-133a contains target sites in the 3′-UTR sequence of the 4 DENV serotypes and that overexpression of miR-133a in Vero cells was associated with decreased DENV-2 replication activity [84]. All these data suggest a possible antiviral mechanism via miR-133a targeting the PTB protein mRNA and the DENV 3′-UTR sequence. Furthermore, we also showed that miR-744 and miR-484 can downregulate DENV replication by targeting the 3′UTR of the DENV RNA genome [Betancur et al., submitted]. In addition, the cellular miR-548g-3p has been identified as displaying antiviral activity by targeting the 5′-UTR SLA (Stem Loop A) promoter of the four DENV serotypes, thus, repressing viral replication and expression of viral proteins, independently of interferon signaling [85]. Moreover, overexpression of miR-223 inhibited replication of DENV in an endothelial cell-like cell line. The authors showed that miR-223 inhibits DENV by negatively regulating the microtubule destabilizing protein stathmin 1 (STMN-1) that is crucial for reorganization of microtubules and later replication of the virus. In addition, this study identified that the transcription factors C/EBP-*α* and EIF2 are regulators of miR-223 expression after DENV infection [86].

Although little is known regarding the variations in miR expression in DENV-infected individuals, a recent study showed the expression profile of the miRs in blood samples of DEN-infected patients. The authors report 12 miRs that were specifically altered upon acute dengue and 17 miRs that could potentially be associated with specific dengue-related complications [164]. In addition, another profiling study reported abundance changes in the expression of some miRs in DENV-infected peripheral blood monocytes. Importantly, let-7e was among the miRs with the most significant regulation which, besides anti-DENV activity, may be of crucial importance for the modulation of inflammatory responses. Specifically, let-7e shares matching sequences with the 3′UTR mRNA of IL-6 and CCL3, as well as of other cytokines, highlighting a key role of miRs in immune response homeostasis during DENV infection (Figure 1) [67, 73, 86]. Likewise, miR-223 that also shares antiviral activity against DENV has been shown to have an important effect on the inflammatory response by regulating IL-*β* and IL-6 through IKK*α* and MKP-5 [86, 165, 166], stressing its potential contribution in DENV pathogenesis control. Since a link between vitamin D and miR expression has been established, but no reports discuss their combined implications for DENV antiviral and inflammatory response, we hypothesized here a vitamin D and miR interplay that could modulate DENV pathogenesis, opening new horizons in the therapeutic field of dengue disease.

## 5. Concluding Remarks and Future Perspectives

Severe dengue disease symptoms and DENV infection are characterized by overproduction of proinflammatory cytokines driven mainly by activation of several PRRs [29]. Here, we hypothesize that vitamin D may contribute to avoiding DENV infection and disease progression, especially through the modulation of miRs/TLRs that enhance the antiviral activity and regulate the inflammatory response. Although vitamin D's antiviral mechanism has not been fully elucidated, it may be linked to vitamin D's ability to control the permissiveness of DENV target cells and the virus-induced proinflammatory responses [72]. However, a better understanding of these mechanisms is required to provide interesting clues regarding DENV pathogenesis and dengue disease treatment. Certainly, epidemiological and experimental evidence describe an overall positive vitamin D-related immune effect in which increased levels of vitamin D and variants in the VDR receptor are associated with reduction of viral replication, decreased risk of infection, lower disease severity, and better outcome of the dengue symptoms [9–12, 72]. Additionally, the emerging relationships between vitamin D, the TLR signaling pathway, and its regulation by miRs are beginning to gain critical importance in infectious diseases. Indeed, as discussed above, several DENV infection studies have started to illustrate these vitamin D regulatory features that could be key mechanisms for the control of virus replication and homeostasis of the inflammatory response, thus making this hormone a special candidate for therapeutic strategies [127]. Although most of the studies have focused on the effects of vitamin D induced in dendritic cells and macrophages, others have also described the same immunoregulatory effects on other cell populations of the immune system such as CD8^+^ T cells, NK cells, and B cells [167–169] suggesting their impact not only on DENV target cells but also at the level of cells associated with virus clearance. All the data discussed here suggest that vitamin D could constitute a strong potential strategy to modulate the “cytokine storm” that occurs during ongoing DENV infections and the progression to severe states of the disease. Although it is important to note that such a global effect on the inflammatory activity could weaken the host response to other opportunistic pathogens, it has been suggested that while vitamin D may reduce inflammatory markers during viral infections, it also exerts protective effects against coinfections with other opportunistic pathogens [14, 106]. Moreover, its clinical effectiveness has been tested by improving the overall physical condition of DENV patients and reducing the progression of the disease [11]. Although incoming supplementary trials are required to fully elucidate the therapeutic relevance of vitamin D, it is evident that this hormone may be an excellent alternative of a natural immune-regulatory agent capable of modulating the innate immune response against DENV, which will provide crucial information to understand and design strategies to treat and control progression of dengue disease. Although further experimental studies are required to boost the understanding of vitamin D in the regulation of inflammation and antiviral response against DENV infection, the information discussed above highlights the features of vitamin D in immune regulation as an exciting research field and as an efficient and low-cost therapeutic procedure against DENV and possibly other viral infections.

## Figures and Tables

**Table 1:** Summary of the main cytokines associated with development of DHF/DSS and their biological function in relation to pathogenesis.

Cytokines	Biological function	Refs.
MCP-1	Monocyte chemoattractant protein-1 is critical to drive the extravasation of mononuclear cells into the inflamed, infected, and traumatized sites of infection. In addition, it promotes endothelial permeability increasing the vascular leakage as a result of dengue virus infection.	[47, 48]

IL-1	It induces tissue factor (TF) expression of endothelial cells (EC) and suppresses their cell surface anticoagulant activity. It may upregulate TNF-*α* production and activity. IL-1*β* mediates platelet-induced activation of ECs, which increases chemokine release and upregulates VCAM-1 enhancing adhesion of monocytes to the endothelium.	[43, 49]

IL-6	It has been described as a strong inducer of endothelial permeability resulting in vascular leakage. IL-6 potentiates the coagulation cascade and can downregulate production of TNF-*α* and its receptors. IL-6 may perform a synergistic role with some pyrogens such as IL-1 to induce fever.	[50, 51]

IL-8	Its systemic concentrations are increased by EC damage, which in turn induces endothelial permeability. Activation of the coagulation system results in increased expression of IL-6 and IL-8 by monocytes, while the APC anticoagulation pathway downregulates the production of IL-8 by ECs.	[49, 50, 52]

IL-10	It plays an immunosuppressive role that causes IFN resistance, followed by impaired immune clearance and a persistent infectious effect for acute viral infection. IL-10 also inhibits the expression of TF and inhibits fibrinolysis. IL-10 plasma levels have been associated with disease severity; however, its role in dengue pathogenesis has not been fully elucidated.	[53]

TNF-*α*	It is a potent activator of ECs; it enhances capillary permeability. TNF-*α* upregulates expression of TF in monocytes and ECs and downregulates expression of thrombomodulin on ECs. It also activates the fibrinolytic system and enhances expression of NO mediating activation-induced death of T cells, and it has therefore been implicated in peripheral T-cell deletion.	[49, 51, 54]

TGF-*β*	Early in infection, low levels of TGF-*β* may trigger secretion of IL-1 and TNF-*β*. However, later in infection, the cytokine inhibits the Th1 response and enhances production of Th2 cytokines such as IL-10. TGF-*β* increases expression of TF on ECs and upregulates expression and release of PAI-1 (plasminogen activator inhibitor-1).	[3]

VEGF	VEGF is a key driver of vascular permeability. It reduces EC occludins, claudins, and the VE-cadherin content, all of which are components of ECs junctions. Upon activation, VEGF stimulates expression of ICAM-1, VCAM-1, and E-selectin in ECs.	[3, 36]

**Table 2:** Vitamin D-induced mechanisms/mediators associated with antiviral activity.

Mediator/mechanism	Virus	Refs.
Cathelicidin (LL-37)	VHS, influenza virus, HIV, retrovirus	[55–59]
HBD2	HIV	[60]
ROS	HCV	[61]
IFN response	HIV, HCV	[62–64]
Autophagy	HIV	[65, 66]
miR let-7	DENV	[67, 68]

**Table 3:** Vitamin D and miR targets associated with inflammatory response.

Target/mediator	Modulator	Refs.
TLR2/4	Vitamin D/miR155.miR146	[20, 69, 70]
TNF-*α*	Vitamin D/miR146	[70, 71]
IL-1*β*	Vitamin D/miR155	[19, 69]
IL-6	Vitamin D/let-7e	[72, 73]
MAPK	Vitamin D	[19]
NF-*κ*B	Vitamin D/miR155, miR146	[20, 70, 74, 75]
IKK	Vitamin D	[76]
SOCS1	Vitamin D/miR155	[20]
TLR9	Vitamin D	[77]

**Table 4:** Summary of miRs regulating DENV-induced inflammatory response and viral replicative activity.

miRNA	Target	Cell line	Refs.
let-7e	3′-UTR of IL-6	Human peripheral blood mononuclear cells	[73]
let-7c	HO-1 protein and the transcription factor BACH1	Huh-7 human hepatic cell line	[68]
miR-252	DENV envelope E protein	*Aedes albopictus* C6/36 cell line	[78]
miR-30e^*∗*^	IkB*α* in DENV-permissive cells and IFN-*β* production	Peripheral blood mononuclear cells and U937 and HeLa cell lines	[79]
miR-150	3′-UTR of SOCS-1	Peripheral blood mononuclear cells and monocytes	[80]
miR-122	3′-UTR of the DENV genome/mRNA	BHK-21, HepG2, and Huh-7 cell lines	[81]
miR-142	3′-UTR of the DENV genome/mRNA	Human dendritic cells and macrophages	[82]
miR-133a	3′-UTR of PTB; 3′-UTR of the DENV genome/mRNA	Mouse C2C12 cells and Vero cells	[83, 84]
miR-548	5′-UTR SLA (Stem Loop A) DENV	U937 monocyte/macrophages	[85]
miR-223	Microtubule destabilizing protein stathmin 1 (STMN-1)	EA.hy926 endothelial cell line	[86]
